# Foliar-Selenium-Induced Modulation of Volatile Organic Compounds in Rice Grains: A Comparative Study of Sodium Selenite and Nano-Selenium

**DOI:** 10.3390/foods14193399

**Published:** 2025-09-30

**Authors:** Yin Xiong, Yingying Hu, Ruomeng Li, Haoyue Cheng, Yulin Wu, Xuhong Tian, Yibo Chen, Jingbo Zhou, Lei Zhao, Chongrong Wang

**Affiliations:** 1 School of Modern Industry for Selenium Science and Engineering, Wuhan Polytechnic University, Wuhan 430023, China; yxiong1988@whpu.edu.cn (Y.X.);; 2 Key Laboratory of Genetics and Breeding of High-Quality Rice in Southern China (Co-Construction by Ministry and Province), Ministry of Agriculture and Rural Affairs, Guangdong Key Laboratory of New Technology in Rice Breeding, Rice Research Institute, Guangdong Academy of Agricultural Sciences, Guangzhou 510640, China

**Keywords:** rice, aroma, volatile organic compounds, HS-SPME-GC-MS, selenium

## Abstract

Rice aroma is influenced by many factors, including selenium (Se) fertilizer. In this study, we investigated the effects of different Se species on the volatile organic compounds (VOCs) in three indica rice varieties over 2022 and 2023 by forliar spray. The VOCs were analyzed using HS-SPME-GC-MS. The results showed that both Se nanoparticles (SeNPs) and sodium selenite (Na_2_SeO_3_) significantly increased the contents of most VOCs in all three varieties, with SeNPs exhibiting a more pronounced effect. PCA and OPLS-DA revealed distinct clustering of the VOCs based on Se treatments and rice varieties. By variable importance in projection (VIP) analysis with FDR correction, Na_2_SeO_3_ yielded 7 markers, whereas SeNP treatment identified 18. Every marker detected under Na_2_SeO_3_ was fully encompassed within the SeNPs set. Three-factor ANOVA indicated that there are significant interaction effects among Se species, rice variety, and planting year. Additionally, the effect sizes were evaluated in the key VOCs to quantify the effect of Se species, rice variety, and planting year. The findings highlight Se fertilizers to enhance rice aroma and suggest selecting appropriate Se species and rice varieties for aroma improvement.

## 1. Introduction

Rice is a staple food for more than half of the world’s population. With the continuous development of the economy, the demand of consumers for rice has shifted from a single pursuit of yield to higher quality and nutrition. Aroma is an important attribute of rice quality, and also an important indicator in flavor evaluation and a key factor affecting the commercial value of rice [1]. Rice aroma is a comprehensive agronomic trait affected by many factors such as genetic background, storage conditions, cultivation modes, and processing methods [2]. Rice aroma is composed of many volatile organic compounds (VOCs), such as aldehydes, alcohols, ketones, and heterocyclic compounds. To date, more than 200 VOCs have been reported in rice [3,4,5]. It has been reported that 2-acetyl-1-pyrrorine (2-AP), 4-vinylphenol, decanal, nonanal, (E)-2-nonenal, octanal, hexanal, 4-vinyl-guaiacol, and (E,E)-2,4-decadienal are the main VOCs contributing to rice aroma [6]. In addition, 2-butoxy ethanol, 1-pentanol, n-octanol, benzyl alcohol, and 2-AP are VOCs that are particularly abundant in aromatic rice [7].

Selenium (Se) is an indispensable trace element for humans, which can improve the immune system and prevent a series of health problems such as Keshan disease and Kaschin–Beck disease [8]. Se cannot be synthesized by the human body, and can only be supplemented through food and exogenous supplements. It has been reported that the daily intake of Se per capita in China is far lower than the 40 μg per day recommended by the World Health Organization [9,10]. Considering that rice provides up to 80% of the daily caloric intake of more than half of the world’s population, increasing the content of Se in rice can effectively solve the problem of dietary Se deficiency. Exogenous application of Se fertilizer is widely employed to produce Se-rich rice. Numerous studies have demonstrated that the concentration and species of Se fertilizers have great impacts on rice quality and Se uptake efficiency [11,12,13]. The main Se species in Se fertilizers include selenite, selenate, Se nanoparticles (SeNPs), and selenomethionine [14]. A study showed that SeNPs could also be taken up by plants and then transformed into organic Se compounds, selenite, and selenate in roots and shoots, and SeNPs that were absorbed by the root would be rapidly converted into organic Se compounds, thus affecting their retention in the root [15]. It has been reported that SeNPs are a type of slow-release Se with lower Se-enrichment efficiency than other Se species [16]. Moreover, it has been suggested that different rice varieties show various characteristics under Se biofortification [17,18].

Currently, genetic breeding and cultivation are the two main pathways for improving rice aroma [19]. It has been shown that exogenous application of Se can improve the synthesis of 2-AP, a key aromatic compound with a “nutty” and “popcorn-like” aroma in rice grains [20,21]. Some studies have evaluated the effect of exogenous application of Se on the rice aroma “fingerprint” through headspace gas chromatography–ion mobility spectrometry (HS–GC–IMS), a recent technique that combines GC and IMS to obtain VOC profiles based on the drift time differences in ionized VOCs in a drift tube under atmospheric pressure [22,23]. HS–GC–IMS is preferred to detect low-boiling-point compounds of C2–C10 molecular weights due to the direct headspace injection, making it difficult to detect VOCs with larger molecular weights or higher boiling points in food. Headspace solid phase microextraction–gas chromatography–mass spectrometry (HS–SPME–GC–MS) shows greater advantages in qualitative and quantitative analysis of VOCs, but is prone to miss small molecular VOCs due to the high temperature used in GC [24]. The main VOCs contributing to rice aroma comprise 2-AP, aldehydes, heterocyclic compounds, alcohols, and phenols [2]. To date, HS–SPME–GC–MS has been widely used for VOC analysis in rice, while SPME is used for VOC absorption and enrichment, GC is used for separation, and MS is used for qualitative and quantitative analysis.

The effects of different Se fertilizers with various Se species on the composition of flavor compounds in rice remain largely unknown. In this study, we conducted a continuous two-year field experiment by supplying three indica rice varieties with SeNPs and Na_2_SeO_3_, aiming to explore how different Se species affect rice aroma and the stability of the effects, and which VOCs can serve as markers of Se treatments, in both aromatic and non-aromatic rice varieties. The experiment involved one aromatic rice variety, Mei xiang zhan (MXZ), and two non-aromatic rice varieties, Huang hua zhan (HHZ) and Huang guang nong zhan (HGNZ). We investigated the VOC profiles of the brown rice and carried out qualitative and quantitative analyses on the VOCs through HS–SPME–GC–MS, and employed a three-factor variance analysis to compare the effects of Se species, rice varieties, and planting years and revealed their interaction effects on rice aroma. The findings are expected to provide new information for the effects of different Se species and the stability of the effects on rice aroma, which is of great significance for the production of high-quality rice.

## 2. Results and Discussion

### 2.1. Qualitative and Quantitative Analysis of VOCs in Brown Rice of Different Varieties Under Various Se Treatments in Two Years

Table 1 shows the VOCs identified in the brown rice of HHZ, MXZ, and HGNZ under three treatments (CK, Na_2_SeO_3_, and SeNPs) in 2022 and 2023. A total of 51 VOCs were identified in the three varieties in 2022, which belonged to aldehydes (13), ketones (12), alcohols (15), esters (2), alkanes (3), and others (6). In 2023, a total of 50 VOCs were identified, including aldehydes (13), ketones (12), alcohols (14), esters (2), alkanes (3), and others (6). Among them, 46 VOCs were detected in all the three varieties over the two years (Table 1). 2-AP and 2-pyrrolidinone were specifically detected in aromatic rice MXZ. Alpha-Terpineol was uniquely found in HGNZ in 2022. Benzaldehyde, (Z)-2-[(2R,5S)-5-Methyl-5-vinyltetrahydro-2-furanyl]-2-propanol and 2-Furanmethanol, 5-ethenyltetra were detected in all varieties in 2022, but only in MXZ in 2023. Hexanoic acid was specifically detected in 22MXZ, 23MXZ, and 23HGNZ. 2-phenyl-2-propanol was specifically detected in 22HHZ, 22MXZ, 22HGNZ, 23HGNZ, and 23MXZ. These results indicated that different rice varieties have different aromatic profiles, which are affected by planting years and different Se treatments.

**Figure 1:**
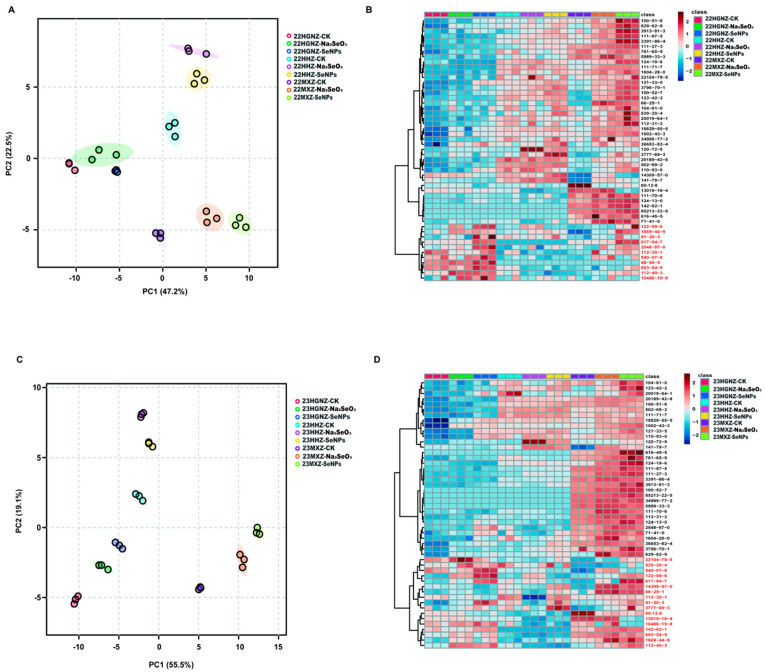
PCA and hierarchical cluster analyses. (**A**). PCA analysis of samples in 2022. (**B**). Heatmaps of VOCs detected in HHZ, HGNZ, and MXZ under different Se treatments in 2022. (**C**). PCA analysis of samples in 2023. (**D**). Heatmaps of VOCs detected in HHZ, HGNZ, and MXZ under different Se treatments in 2023. The shadow in subfigure A and C refer to the 95% confidence regions.

To evaluate the consistency of each sample and explore the influence of different Se and CK treatments on HHZ, HGNZ, and MXZ, a PCA analysis was conducted on the data of 2022 and 2023, respectively. In 2022, the first principal component (PC1, 47.2%) and second principal component (PC2, 22.5%) accounted for 69.7% of the total variance (Figure 1A). Within each rice variety, the CK, Na_2_SeO_3_, and SeNP treatments were spatially separated, indicating that exogenous application of different Se species has specific impacts on the VOCs in rice. To further verify the results and evaluate the effects of different Se species on the three varieties, hierarchical cluster analyses were performed according to the contents of all VOCs in each sample in 2022. A heatmap was used to visualize the differences in compound concentrations across treatments and varieties. The intensity of the color in each cell of the heatmap indicates the concentration level of the corresponding VOCs (Figure 1B). The color scale was typically calibrated so that blue shades represent lower concentrations, while red shades represent higher concentrations. Obviously, the three rice varieties showed different VOC profiles. Longitudinally, the VOCs detected in 2022 could be classified into group A (40 VOCs, marked with black) and group B (11 VOCs, marked with red) (Figure 1B). HGNZ had relatively higher concentrations of VOCs in group B, while HHZ had relatively high concentrations of certain VOCs in group A and lower concentrations of most VOCs in group B. Furthermore, MXZ, a typical aromatic rice variety, had higher concentrations of most VOCs in both group A and group B compared with the other two non-aromatic varieties. In general, both Na_2_SeO_3_ and SeNP treatments could increase the contents of most VOCs in all the three varieties compared with the CK treatment.

In 2023, PC1 (55.5%) and PC2 (19.1%) together accounted for 74.6% of the total variance (Figure 1C). Consistent with the results in 2022, HHZ, HGNZ, and MXZ were divided into three different categories in 2023, and both Na_2_SeO_3_ and SeNP treatments were separated from CK in all varieties. These results suggested that different rice varieties have various VOC profiles, and application of both Na_2_SeO_3_ and SeNPs can lead to genotype-dependent changes in the VOC profiles in rice grains. A vertical observation of the heatmap showed that the patterns in the contents of VOCs under both Na_2_SeO_3_ and SeNP treatments were similar to those observed in 2022 (Figure 1D). The VOCs could be classified into group A (33 VOCs, marked in black) and group B (17 VOCs, marked in red) as well. Consistent with the VOCs detected in 2022, HGNZ showed much higher concentrations of the VOCs in group B than those in group A, and nine VOCs in group B in 2022 were overlapped with the VOCs in group B in 2023. In addition, HHZ showed a different aromatic profile and the concentrations of most VOCs in MXZ were higher than those in non-aromatic rice varieties HHZ and HGNZ.

### 2.2. Influence of SeNPs and Na_2_SeO_3_ on VOCs in Three Rice Varieties

To compare the influence of SeNPs and Na_2_SeO_3_ on the three rice varieties, a multiple comparison analysis was conducted among three treatments (CK, Na_2_SeO_3_, and SeNPs) in 2022 (Table S1) and 2023 (Table S2).

Here, how many VOCs were affected by SeNP and Na_2_SeO_3_ treatments were compared, respectively. For MXZ, 50 VOCs were detected under the three treatments in 2022. SeNP treatment led to significantly higher contents of thirty-seven VOCs compared with CK and twenty-one VOCs compared with Na_2_SeO_3_ treatment, respectively. Meanwhile, Na_2_SeO_3_ treatment significantly increased the levels of twenty-seven VOCs relative to the control (CK), and only three VOCs relative to the SeNP treatment. Among these significantly increased VOCs, the key VOC was 2-AP, in aromatic rice, whose content was 38.6% and 17.2% higher than that under CK and Na_2_SeO_3_ treatment, respectively. In 2023, 50 VOCs were detected in MXZ under the three treatments. SeNP treatment significantly increased the contents of thirty-nine and twenty VOCs compared with CK and Na_2_SeO_3_ treatment, respectively, while Na_2_SeO_3_ treatment significantly increased the contents of thirty-three and ten VOCs compared with CK and SeNP treatment, respectively, including 2-AP, whose content was increased by 25.6% and 13% by SeNP treatment relative to that under CK and Na_2_SeO_3_ treatment, respectively. These results indicated that both SeNP and Na_2_SeO_3_ treatments can significantly improve the aroma of aromatic rice varieties, with SeNPs showing a more pronounced effect.

For HHZ, 46 VOCs were detected in 2022. SeNP treatment resulted in significantly higher contents of fifteen and eleven VOCs compared to CK and Na_2_SeO_3_ treatments, respectively, while Na_2_SeO_3_ treatment resulted in significantly higher contents of eight and three VOCs compared to CK and SeNP treatments, respectively. In 2023, 44 VOCs were detected. SeNP treatment resulted in significantly higher contents of fourteen and sixteen VOCs compared to CK and Na_2_SeO_3_ treatments, respectively, while Na_2_SeO_3_ treatment resulted in significantly higher contents of eight and nine VOCs compared to CK and SeNP treatments, respectively. Among these significantly increased VOCs, many were typical rice aroma substances, such as 1-hexanol, 2-pentyl-furan, benzaldehyde, vanillin, and other VOCs with a positive contribution to rice flavor.

For HGNZ, 48 VOCs were detected in 2022. SeNP treatment led to significantly higher contents of twenty six and nine VOCs compared to CK and Na_2_SeO_3_ treatments, respectively. In addition, compared with CK and SeNP treatment, Na_2_SeO_3_ treatment significantly elevated the levels of fifteen and two VOCs. In 2023, 45 VOCs were detected. SeNP treatment significantly increased the content of twenty-eight and nineteen VOCs compared with CK and Na_2_SeO_3_ treatments, respectively. Compared with CK and SeNP treatment, Na_2_SeO_3_ treatment significantly elevated the levels of eighteen and five VOCs. Among these significantly increased VOCs, many were typical rice aroma substances, such as (E)-2-heptenal, benzaldehyde, benzyl alcohol, (E)-6,10-Dimethylundeca-5,9-dien-2-one, and vanillin.

The above results revealed that exogenous Se application could not only enhance the aroma of aromatic rice but also increase the content of VOCs in non-aromatic rice varieties, and the effect is more pronounced in aromatic rice varieties. Furthermore, SeNP application was more effective to increase the VOC contents than Na_2_SeO_3_ application.

**Figure 2:**
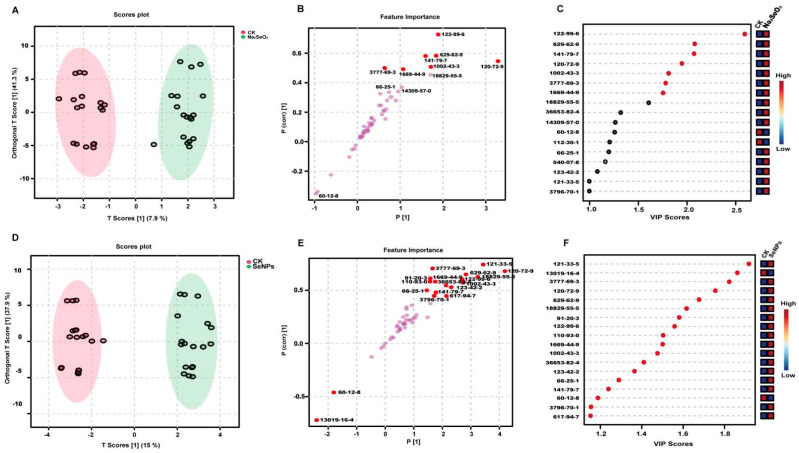
Comprehensive statistical analysis of VOCs. (**A**). OPLS-DA score plot of rice samples from different rice varieties and years between CK and Na_2_SeO_3_ treatments. (**B**). S-plot of rice samples from different rice varieties and years between CK and Na_2_SeO_3_ treatments, R^2^Y = 0.968, Q^2^ = 0.943 (1000 permutation, *p* < 0.001). (**C**). Important volatiles (VIP > 1.0) identified by OPLS-DA of rice samples from different rice varieties and years between CK and Na_2_SeO_3_ treatments. (**D**). OPLS-DA score plot of rice samples from different rice varieties and years between CK and SeNP treatments. (**E**). S-plot of rice samples from different rice varieties and years between CK and SeNP treatments, R^2^Y = 0.989, Q^2^ = 0.973 (100 permutation, *p* < 0.001). (**F**). Important volatiles (VIP > 1.0) identified by PLS-DA of rice samples from different rice varieties and years between CK and SeNP treatments. The shadow in subfigure A and D refer to the 95% confidence regions.

**Figure 3:**
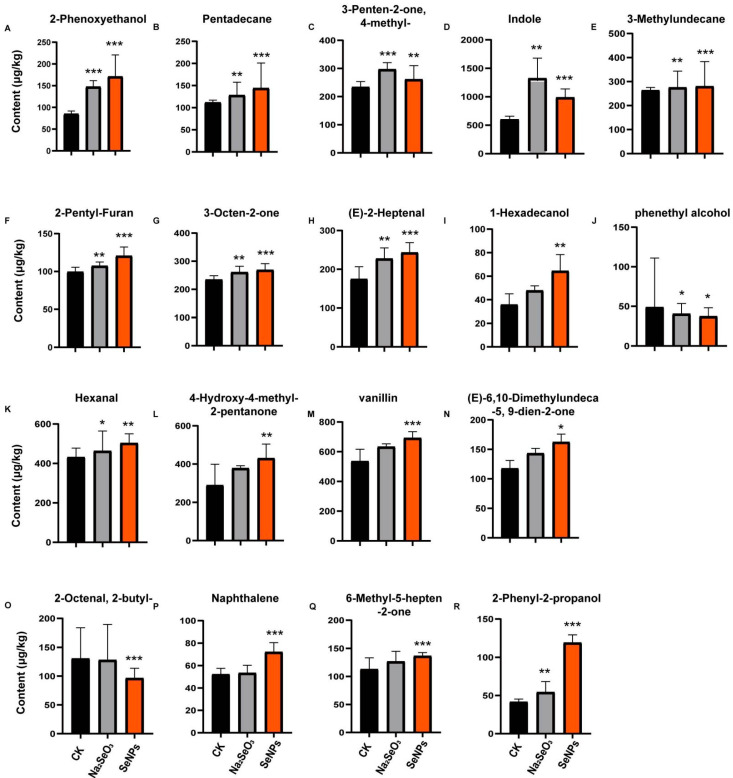
Bar graphs of relative concentrations of the VOCs with VIP scores above 1 in the OPLS-DA of CK-Na_2_SeO_3_ and CK-SeNPs; the relative concentrations are presented in the y-axis (μg/kg): 2-Phenoxyethanol (**A**); Pentadecane (**B**); 3-Penten-2-one, 4-methyl- (**C**); Indole (**D**); 3-Methylundecane (**E**); 2-Pentyl-Furan (**F**); 3-Octen-2-one (**G**); (E)-2-Heptenal (**H**); 1-Hexadecanol (**I**); phenethyl alcohol (**J**); Hexanal (**K**); 4-Hydroxy-4-methyl-2-pentanone (**L**); vanillin (**M**); (E)-6,10-Dimethylundeca-5, 9-dien-2-one (**N**); 2-Octenal, 2-butyl- (**O**); Naphthalene (**P**); 6-Methyl-5-hepten-2-one (**Q**); 2-Phenyl-2-propanol (**R**). Each treatment group has *n* = 18, reflecting three biological replicates of three varieties measured over two consecutive years. The boxplots depict the median and 95% confidence interval of three independent experiments for all panels, * *p* < 0.05; ** *p* < 0.01; *** *p* < 0.001 (Dunn’s Test).

An orthogonal partial least squares-discriminant analysis (OPLS-DA) was employed to explore the potential markers for SeNPs and Na_2_SeO_3_ treatments. OPLS-DA is an advanced version of PLS, making it easier to understand the two components and also enabling the evaluation of variance within and between groups [26]. Given that OPLS-DA can only distinguish between two groups, a pairwise comparison of OPLS-DA was conducted using leave-one-out cross-validation. The scatter scores of the analysis among SeNP- and Na_2_SeO_3_-treated rice samples from different rice varieties and years are presented in Figure 2A–C and Figure 2D–F, respectively. The R^2^Y measures how well the model fits the data, whereas the Q^2^ measures the predictive accuracy of the model, with a Q^2^ value above 0.5 representing a robust model [27]. The original model exhibited high explanatory and predictive capacity, with R^2^Y = 0.968, Q^2^ = 0.943 (1000 permutation, *p* < 0.001) for Na_2_SeO_3_ treatment vs. CK (Supplementary Figure S1A), and a cross-validation ANOVA was performed, and the *p*-value of CV-ANOVA was 9.65777 × 10^−15^. For SeNP treatment vs. CK, the R^2^Y = 0.989, Q^2^ = 0.973 (100 permutation, *p* < 0.001), and the *p*-value of the CV-ANOVA was 1.00432 × 10^−19^ (Supplementary Figure S1B), confirming that the model was not over-fitted and performed significantly better than random chance. Additionally, the low R^2^Y and Q^2^ values observed for the orthogonal components (o1–o3) indicate the successful removal of systematic variation unrelated to class separation, further supporting the model’s reliability (Supplementary Figure S1C,D). The S-plot typically displayed the distribution of samples from different treatment groups, such as the samples from SeNPs and CK (Figure 2A) and from Na_2_SeO_3_ and CK (Figure 2D). Each point on the S-plot represents a detected VOC. There was a clear separation between groups, further indicating that the OPLS-DA model could effectively distinguish between the above treatments. In addition, the plot displayed the importance of 51 detected VOCs (p[1] and p(corr)[1]), which typically indicates the contribution of each VOC to the model. The positions of these points along the x-axis (Feature Importance) indicate the importance of each compound in the model. The compounds with a greater distance from the origin are more influential in distinguishing between the SeNP and CK groups (Figure 2B) and between the Na_2_SeO_3_ and CK groups (Figure 2E). The y-axis represents the correlation of each compound with the response variable (treatment effect). The compounds closer to the top or bottom of the plot have stronger correlations with the treatment, suggesting that they are potential biomarkers for the effect of SeNPs and Na_2_SeO_3_. The VIP (variable importance in projection) scores were used to identify compounds with a significant contribution to the model (Figure 2C,F). To identify the most important markers, the concentrations of the VOCs with VIP values greater than 1.0 were pairwise compared between treatments groups and the *p*-values of these VOCs were calculated (Figure 3). The VOCs with VIP scores greater than 1.0 and *p*-values less than 0.05 were considered as significant, and were recognized as the markers that can distinguish the two groups in OPLS-DA models [28]. The dots in the figure represent the screened VOCs (VIP > 1.0, *p*-value < 0.05, adjusted by FDR), which were marked in red (Figure 2B,E). As a result, only 7 VOCs (FDR < 0.05) were identified as markers that could differentiate the CK and Na_2_SeO_3_ treatment (Figure 2C, marked in red dots; Figure 3A–G), while 18 VOCs (FDR < 0.05) could serve as markers for discriminating the CK and SeNP treatment (Figure 2F and Figure 3A–R).

Seven VOCs were identified as markers for both SeNP and Na_2_SeO_3_ treatments (Figure 3A–G). All these VOCs (2-phenoxyethanol; pentadecane; 3-penten-2-one,4-methyl-; indole; 3-methylundecane; 2-pentyl-Furan; 3-octen-2-one) significantly increased after Na_2_SeO_3_ and SeNP treatments. The markers identified under Na_2_SeO_3_ treatment were fully encompassed within those found under SeNP treatment; however, SeNPs revealed 11 additional markers. This outcome further demonstrates that SeNP application is more effective than Na_2_SeO_3_ in enhancing rice aroma. All these results indicated that both Na_2_SeO_3_ and SeNP treatments can improve the richness and intensity of VOCs.

Phenethyl alcohol has a rose-like aroma, and is a significant aromatic component in Baijiu [29]. Interestingly, SeNP treatments suppressed the content of phenethyl alcohol. Naphthalene is a key VOC in japonica rice porridge with a pungent smell [30]. Vanillin and 2-phenoxyethanol were marker VOCs with the highest VIP for SeNP treatment and Na_2_SeO_3_ treatment, respectively. Vanillin exhibits a vanilla-like odor while 2-phenoxyethanol has a pleasant aroma.

In recent years, an increasing number of studies have shown that exogenous selenium biofortification can enhance the aroma of crops [31,32,33]. However, research on rice fragrance has largely focused on fragrant rice varieties and the key aroma compound 2-acetyl-1-pyrroline (2-AP). The present study reveals that exogenous SeNPs and Na_2_SeO_3_ treatments influence rice aroma through both shared and distinct metabolic mechanisms. In summary, the results here suggested that SeNP and Na_2_SeO_3_ treatments have similar metabolic mechanisms to regulate the VOCs in rice grains, which may be the exact reason for the overlapping of seven VOC markers for SeNP and Na_2_SeO_3_ treatments. However, there were still some differences between SeNP and Na_2_SeO_3_ treatments. For instance, these VOCs had different VIP scores between the two treatments, with 2-phenoxyethanol and vanillin showing the highest VIP score for Na_2_SeO_3_ and SeNP treatment, respectively. These findings further highlight the potential effect of SeNP and Na_2_SeO_3_ treatments on the metabolic mechanisms to affect rice aroma.

### 2.3. Three-Factor ANOVA

To compare the effects of Se species, rice varieties, and planting years and their interaction effects on rice aroma, a three-factor ANOVA was conducted (Table 2). Variety (V) and Se species (Se) showed highly significant effects on nearly all the detected VOCs, as indicated by the high F-values and *p*-values, suggesting that both variety and Se species significantly influence the VOCs. The planting year (Y) also had significant impacts on most compounds, as indicated by the “*” symbol next to the F-values, indicating that the planting year also affects the VOCs in rice grains. In terms of the one aromatic rice variety and two non-aromatic rice varieties, most VOCs showed significant *p*-values, and the F-values of variety were much higher than those of Se species. For example, 2-AP, one of the key VOCs in aromatic rice, exhibited high F values of variety and planting year; however, the Se species also showed a significant *p*-value, indicating that although variety and planting year are vital factors for 2-AP, Se species also has significant effects on 2-AP. Additionally, the effect sizes (partial η^2^) were evaluated in the 18 marker VOCs to quantify the effect of Se species, rice variety, and planting year (Supplementary Table S3). Among these VOCs, five VOCs (2-pentyl-furan, 3-octen-2-one, naphthalene, 2-phenyl-2-propanol, 2-phenoxyethanol) showed a much larger partial η^2^ value of Se species than that of the variety. Furthermore, all five of these VOCs showed much higher partial η^2^ values of Se species than those of the planting year, indicating that despite the effects of rice variety and planting year, these five VOCs were mainly controlled by Se species. Moreover, 2-pentyl-furan in rice grains exhibits a characteristic nutty aroma at low concentrations, but a less pleasant odor similar to that of soybeans at higher concentrations [34]. Aromatic rice had a much higher content of 2-pentyl-furan compared with non-aromatic rice [35]. 3-octen-2-one exhibits a citrus, floral, green, and fruity odor and 2-phenoxyethanol has a pleasant aroma. Thus, exogenous Se application can improve the richness of rice aroma.

V × Se interaction had significant impacts on many compounds, suggesting that the effect of Se application varies with rice variety. V × Y interaction also had a significant effect, indicating that the response to Se treatment may differ across planting years depending on the rice variety. Se × Y interaction was also significant, implying that the effect of Se application can vary from year to year. The V × Se × Y interaction showed significant impacts for some compounds, indicating that the variety, Se species, and planting year have a synergistic significant influence on the VOCs. Many VOCs were significantly affected by individual factors and interaction effects of multiple factors, suggesting complex relationships between these factors. These results suggested that although the variety and planting year have certain impacts on the VOCs, the application of different types of Se has greater and stable effects on some VOCs in rice. The response of different rice varieties to exogenous Se application is influenced by a combination of factors, including variety characteristics, environmental conditions, and Se application methods, which need to be fully considered to achieve the best comprehensive rice quality.

## 3. Materials and Methods

### 3.1. Field Experiment Design

The field experiment was conducted at the experimental farm of the Guangdong Academy of Agricultural Sciences in Guangzhou, Guangdong Province in the summers of 2022 and 2023. Three elite inbred indica rice varieties bred by the Guangdong Academy of Agricultural Sciences were used for Se application, among which MXZ is an aromatic rice variety while HGNZ and HHZ are non-aromatic rice varieties. The experiment was arranged in a randomized complete block design with three replications. Every replicate area was 10 m^2^. The planting spacing was 18.5 cm × 16.5 cm, with a single seedling per hill. Five border rows were planted around the edges as guard rows. (The field block structure refers to the schematic diagram in Supplementary Figure S2).

Total Nitrogen (N) 8.76 kg/666.7 m^2^, ratio 1 N: 0.6 P_2_O_5_: 1.2 K_2_O. 40% basal, 20% mid-tillering, 30% at first panicle differentiation, 5–10% heading; Phosphorus (P) all basal; Potassium (K) split tillering and panicle differentiation. Keep 3–4 cm water recovery, shallow tillering; drain at 80–90% stems, re-flood two-leaf pre-heading, alternate wet/dry post-heading, drain 7 days pre-harvest. The average temperature were ranged from 22.9 °C to 31.2 °C in 2022 and 22.8 °C to 30.7 °C in 2023.

The physical and chemical properties of the soil (0–20 cm) were as follows: pH (H_2_O), 5.48; electrical conductivity, 2.07 × 10^−2^ s/m; organic matter, 42.88 g/kg; total N, 2.33 g/kg; total P, 0.98 g/kg; total K, 8.83 g/kg; total Se, 1.49 mg/kg; total Cadmium (Cd), 0.20 mg/kg; total Arsenic (As), 17.74 mg/kg; total lead (Pb), 53.25 mg/kg; total chromium (Cr), 81.59 mg/kg; total Zinc (Zn), 18.39 mg/kg; total Copper (Cu), 30.59 mg/kg.

### 3.2. Se-Rich Cultivation of Different Rice Varieties

In this study, SeNPs were synthesized with ascorbic acid and Na_2_SeO_3_ according to the previously reported method with some modifications [36]. Sodium starch octenyl succinate (SSOS) was used as the template to maintain the stability and bioactivity of SeNPs. To block the effect of SSOS background on rice aroma, all blank tests and Na_2_SeO_3_ treatments were added with the same dose of SSOS in the SeNP treatments. The treatments included CK (control, 0.4 g/L SSOS); Na_2_SeO_3_ (0.03 g/L Na_2_SeO_3_ with 0.4 g/L SSOS); and SeNPs (0.014 g/L SeNPs with 0.4 g/L SSOS). The concentration of Se delivered to the leaf both in Na_2_SeO_3_ treatments and SeNPs were 14 mg/L. In both 2022 and 2023, the diameters of spherical SeNPs consistently fell within the 20–100 nm range according to the scanning electron microscopy. Each treatment was performed with three replicates in a completely randomized block design on HHZ, HGNZ, and MXZ, respectively. SeNPs and Na_2_SeO_3_ were sprayed on the leaves of rice at the heading stage with the same dose of Se (7 g/ha). The grains from different treatments were harvested 40 days after heading and then stored in a stocking chamber for one month to equilibrate the rice grain moisture to 14%, and then the aroma evaluation was conducted. The total Se content was tested and a series of Se-rich rice of different varieties were generated, including MXZ-CK (0.113 ± 0.001/0.118 ± 0.011 mg/kg); MXZ-SeNPs (0.174 ± 0.001/0.322 ± 0.013 mg/kg); MXZ-Na_2_SeO_3_ (0.389 ± 0.007/0.701 ± 0.023 mg/kg); HHZ-CK (0.099 ± 0.007/0.134 ± 0.005 mg/kg); HHZ-SeNPs (0.164 ± 0.001/0.327 ± 0.012 mg/kg); HHZ-Na_2_SeO_3_ (0.361 ± 0.002/0.434 ± 0.026 mg/kg); HGNZ-CK (0.128 ± 0.002/0.148 ± 0.015 mg/kg); HGNZ-SeNPs (0.172 ± 0.002/0.298 ± 0.051 mg/kg); HGNZ-Na_2_SeO_3_ (0.313 ± 0.010/0.615 ± 0.006 mg/kg). Data were shown as total Se contents in 2022 and 2023, respectively.

### 3.3. HS-SPME-GC-MS Analysis

In both 2022 and 2023, the harvested rice seeds were equilibrated in an ultra-low humidity electronic dry cabinet (iHZMs-200, Mingri Baiao, Beijing, China) with humidity maintained at <25% RH for one month to achieve a constant moisture of 14%. First, the seeds were dehulled to produce brown rice, and then healthy and plump brown rice were selected and ground and then the brown rice powder was passed through a 60-mesh sieve. A total of 5 g of brown rice powder from each sample was weighed and placed in a 20 mL headspace bottle containing 20 μL of internal standard substance (2,4,6-trimethylpyridine, diluted with methanol) with a mass concentration of 0.0125 μg/μL, and sealed with a polytef spacer. The solid-phase microextraction sampling conditions were as follows. The headspace bottle was incubated in equilibrium at 80 °C for 10 min; the extraction head was inserted and adsorbed for 40 min; and the samples were desorbed at the inlet at 250 °C for 5 min, and then analyzed by GC-MS (EXPEC 5231, Hangzhou Puyu Instrument Co., Ltd., Hangzhou, China). The SPME fiber used for GC–MS analysis was a Smart SPME Fiber DVB/C-WR/PDMS 80/10-P1 (dark gray), manufactured by a PAL System, Zwingen, Switzerland (part no. 2022139-0). The column was DB-WAX capillary column (Agilent J&W, 30 m × 0.320 mm, 0.25 μm). The carrier gas was helium with a flow rate of 1.8 mL/min, and the sample was not divided. The heating procedure was to keep the initial temperature at 40 °C for 2 min, rise to 120 °C at 3 °C/min, to 190 °C at 5 °C/min, and then to 230 °C at 10 °C/min for 5 min. The mass spectrum conditions were an electron ionization source, an electron energy of 70 eV, a transmission line temperature of 250 °C, an ion source temperature of 230 °C, and a mass scanning range of 50~400 *m*/*z*. The NIST 11 spectrum library was used for searching and the retention index (RI) was used as the index to report the identification results of the mass spectrum matching index greater than 800. If it was less than 800, the RI values of the corresponding substances were compared with those in the literature. The retention times of n-alkanes ranging from C9–C26 were analyzed, and the RI was calculated by the formula RI(X)= [(log tR(x) − log tR(z)) ÷ (log tR(z + 1) − log tR(z)) + Z] × 100, where tR(x), tR(z), and tR(Z + 1) are the retention time of an unknown compound x and normal alkanes with carbon atom numbers of Z and Z + 1, respectively [33]. The peak of compound x would appear after that of a normal alkane with Z carbon atoms and before that of a normal alkane with Z + 1 carbon atoms. The ratio of the peak area of each component to the peak area of the internal standard (2,4,6-trimethylpyridine) was used as the relatively quantitative standard. The relative contents of VOCs were calculated with the following formula: Content (μg/kg) = unknown compound ion peak area ÷ internal standard ion peak area × 0.25 μg (internal standard mass) ÷ 5 g (total sample mass) × 1000.

### 3.4. Statistic Analysis

All treatments were conducted in triplicate. PCA is a statistical analysis method to simplify data and highlight the inter-relationship between different samples, and is widely used in sample variation analysis [37]. For PCA and OPLS-DA analysis the and heatmap cluster, the data were normalized by square root transformation and Pareto scaling (mean-centered and divided by the square root of the standard deviation of each variable). The PCA and OPLS-DA were conducted online (https://www.metaboanalyst.ca/, accessed on 22 May 2025). The CV-ANOVA were analyzed by SIMCA 14.1. The Dunn’s test, three-factor variance analysis, and effect size evaluation were performed by R studio (version 4.2.1).

## 4. Conclusions

In this study, PCA and hierarchical cluster analysis revealed clear differences in VOC profiles among treatments and varieties. Then, OPLS-DA was used to identify specific VOC markers for each treatment, highlighting the effect of Se to regulate VOC metabolic mechanisms to affect rice aroma. Additionally, three-factor ANOVA confirmed significant effects of rice variety, Se species, planting year, and their interaction effects on VOCs. The results demonstrated that both Na_2_SeO_3_ and SeNP treatments significantly increased the content and diversity of VOCs in rice grains, particularly SeNP treatment. Both Na_2_SeO_3_ and SeNP treatments enhanced the aroma of aromatic rice varieties and increased the VOC content in non-aromatic varieties. 2-phenoxyethanol, pentadecane, 3-penten-2-one, 4-methyl-, indole, 3-methylundecane, 2-pentyl-Furan, and 3-octen-2-one are marker VOCs for both Na_2_SeO_3_ and SeNP treatments. Moreover, the three rice varieties exhibited distinct VOC profiles, which are influenced by the Se species and the planting year. In summary, our results indicated that the application of Se, particularly SeNPs, can effectively improve rice aroma, but the response varies depending on the rice variety and environmental conditions. Compared with non-aromatic rice, exogenous Se fortification can more effectively enhance the aroma of aromatic rice grains and among different Se species, SeNPs shows better effects. These findings suggest that optimizing Se application and selecting appropriate rice varieties can effectively enhance rice aroma.

## Figures and Tables

**Table 1:** Qualitative results of volatile organic compounds detected in brown rice generated by different Se treatments of HHZ, MXZ, and HGNZ in 2022 and 2023.

	Compound	CAS	Formula	MW	Match Index	RT (min)	RI	RI (Literature)	Odor Description	Detected Samples
	Aldehydes (13)									
1	Hexanal	66-25-1	C_6_H_12_O	100.2	829	5.85	1078	1080	Fruity, grass, green, green tomato,	22HHZ, 22HGNZ, 22MXZ, 23HHZ, 23HGNZ, 23MXZ
2	Heptanal	111-71-7	C_7_H_14_O	114.2	885	8.8	1180	1182	Grass, fresh, floral, fatty, citrus, rancid	22HHZ, 22HGNZ, 22MXZ, 23HHZ, 23HGNZ, 23MXZ
3	Octanal	124-13-0	C_8_H_16_O	128.2	923	12.51	1280	1286	Citrus, floral, Lemon, green, fat,	22HHZ, 22HGNZ, 22MXZ, 23HHZ, 23HGNZ, 23MXZ
4	(E)-2-Heptenal	18829-55-5	C_7_H_12_O	112.2	873	13.83	1311	1321	Slightly fruity	22HHZ, 22HGNZ, 22MXZ, 23HHZ, 23HGNZ, 23MXZ
5	1-Nonanal	124-19-6	C_9_H_18_O	142.2	879	16.71	1380	1380	Citrus, fatty, orange peel, waxy	22HHZ, 22HGNZ, 22MXZ, 23HHZ, 23HGNZ, 23MXZ
6	(E)-2-Octenal	2548-87-0	C_8_H_14_O	126.2	901	18.02	1410	1412	Green and fatty-like	22HHZ, 22HGNZ, 22MXZ, 23HHZ, 23HGNZ, 23MXZ
7	Decanal	112-31-2	C_10_H_20_O	156.3	892	20.99	1481	1485	Fatty, citrusy, sweet, floral, waxy	22HHZ, 22HGNZ, 22MXZ, 23HHZ, 23HGNZ, 23MXZ
8	Benzaldehyde	100-52-7	C_7_H_6_O	106.1	893	21.54	1493	1508	Nutty, sweet, bitter, almond	22HHZ, 22HGNZ, 22MXZ, 23MXZ
9	2-Methylbenzaldehyde	529-20-4	C_8_H_8_O	120.1	826	25.43	1588	1600	Mild floral, sweet	22HHZ, 22HGNZ, 22MXZ, 23HHZ, 23HGNZ, 23MXZ
10	(E)-2-Decenal	3913-81-3	C_10_H_18_O	154.2	847	26.55	1616	1630	Fatty and waxy-like	22HHZ, 22HGNZ, 22MXZ, 23HHZ, 23HGNZ, 23MXZ
11	2-Octenal, 2-butyl-	13019-16-4	C_12_H_22_O	182.3	904	27.55	1644	1659	-	22HHZ, 22HGNZ, 22MXZ, 23HHZ, 23HGNZ, 23MXZ
12	Tridecanal	10486-19-8	C_13_H_26_O	198.3	852	32.62	1794	1800	-	22HHZ, 22HGNZ, 22MXZ, 23HHZ, 23HGNZ, 23MXZ
13	Vanillin	121-33-5	C_8_H_8_O_3_	152.1	927	49.24	2576	2566	Vanilla-like	22HHZ, 22HGNZ, 22MXZ, 23HHZ, 23HGNZ, 23MXZ
	**Ketones (12)**									
14	3-Penten-2-one, 4-methyl-	141-79-7	C_6_H_10_O	98.1	841	7.15	1127	1125	-	22HHZ, 22HGNZ, 22MXZ, 23HHZ, 23HGNZ, 23MXZ
15	6-Methyl-5-hepten-2-one	110-93-0	C_8_H_14_O	126.2	815	14.46	1328	1336	Herby, green, banana-like	22HHZ, 22HGNZ, 22MXZ, 23HHZ, 23HGNZ, 23MXZ
16	4-Hydroxy-4-methyl-2-pentanone	123-42-2	C_6_H_12_O_2_	116.2	903	15.37	1350	1352	-	22HHZ, 22HGNZ, 22MXZ, 23HHZ, 23HGNZ, 23MXZ
17	3-Octen-2-one	1669-44-9	C_8_H_14_O	126.2	826	17.16	1391	1414	Citrus, floral, green, fruity	22HHZ, 22HGNZ, 22MXZ, 23HHZ, 23HGNZ, 23MXZ
18	2-Decanone	693-54-9	C_10_H_20_O	156.3	844	20.82	1477	1482	Orange-like, fruity, fatty	22HHZ, 22HGNZ, 22MXZ, 23HHZ, 23HGNZ, 23MXZ
19	3-Nonen-2-one	14309-57-0	C_9_H_16_O	140.2	857	21.45	1491	1506	Pleasant fruity	22HHZ, 22HGNZ, 22MXZ, 23HHZ, 23HGNZ, 23MXZ
20	3,5-Heptadien-2-one,6-methyl-	1604-28-0	C_8_H_12_O	124.2	925	24.56	1568	1582	-	22HHZ, 22HGNZ, 22MXZ, 23HHZ, 23HGNZ, 23MXZ
21	2(5H)-Furanone, 5,5-dimethyl-	20019-64-1	C_6_H_8_O_2_	112.1	846	24.94	1577	1590	-	22HHZ, 22HGNZ, 22MXZ, 23HHZ, 23HGNZ, 23MXZ
22	(E)-6,10-Dimethylundeca-5,9-dien-2-one	3796-70-1	C_13_H_22_O	194.3	811	33.82	1836	1849	Rose, floral, fruity	22HHZ, 22HGNZ, 22MXZ, 23HHZ, 23HGNZ, 23MXZ
23	2-Pyrrolidinone	616-45-5	C_4_H_7_NO	85.1	812	38.11	2018	2020	-	22MXZ, 23MXZ
24	2-Pentadecanone,6,10,14-trimethyl-	502-69-2	C_18_H_36_O	268.5	867	40.92	2121	2110	-	22HHZ, 22HGNZ, 22MXZ, 23HHZ, 23HGNZ, 23MXZ
25	3-Ethyl-4-methyl-1H-pyrrole-2,5-dione	20189-42-8	C_7_H_9_NO_2_	139.2	893	44.45	2270	2260	-	22HHZ, 22HGNZ, 22MXZ, 23HHZ, 23HGNZ, 23MXZ
	**Alcohols (15 in 2022, 14 in 2023)**									
26	1-Pentanol	71-41-0	C_5_H_12_O	88.1	885	11.44	1253	1252	Plastic, moderately strong, green, fusel oil-like	22HHZ, 22HGNZ, 22MXZ, 23HHZ, 23HGNZ, 23MXZ
27	1-Hexanol	111-27-3	C_6_H_14_O	102.2	852	15.52	1353	1360	Green, herbaceous, sweet	22HHZ, 22HGNZ, 22MXZ, 23HHZ, 23HGNZ, 23MXZ
28	(Z)-2-[(2R,5S)-5-Methyl-5-vinyltetrahydro-2-furanyl]-2-propanol	5989-33-3	C_10_H_18_O_2_	170.2	851	18.75	1430	1425	-	22HHZ, 22HGNZ, 22MXZ, 23MXZ
29	1-Octen-3-ol	3391-86-4	C_8_H_16_O	128.2	845	19.5	1447	1451	Raw mushroom, straw	22HHZ, 22HGNZ, 22MXZ, 23HHZ, 23HGNZ, 23MXZ
30	1-Heptanol	111-70-6	C_7_H_16_O	116.2	846	19.7	1452	1454	Woody, sweet, green	22HHZ, 22HGNZ, 22MXZ, 23HHZ, 23HGNZ, 23MXZ
31	2-Furanmethanol,5-ethenyltetra	34995-77-2	C_10_H_18_O_2_	170.2	803	19.96	1458	1452	-	22HHZ, 22HGNZ, 22MXZ, 23MXZ
32	1-Octanol	111-87-5	C_8_H_18_O	130.2	916	23.83	1550	1558	Fatty, metallic, citrus, fruity, floral	22HHZ, 22HGNZ, 22MXZ, 23HHZ, 23HGNZ, 23MXZ
33	2-Octen-1-ol	22104-78-5	C_8_H_16_O	128.2	877	26.02	1602	1613	-	22HHZ, 22HGNZ, 22MXZ, 23HHZ, 23HGNZ, 23MXZ
34	alpha-Terpineol	98-55-5	C_10_H_18_O	154.2	803	28.94	1679	1680	-	22HGNZ
35	1-Decanol	112-30-1	C_10_H_22_O	158.3	816	31.39	1754	1752	-	22HHZ, 22HGNZ, 22MXZ, 23HHZ, 23HGNZ, 23MXZ
36	Benzyl alcohol	100-51-6	C_7_H_8_O	108.1	879	34.3	1860	1866	Slightly sweet	22HHZ, 22HGNZ, 22MXZ, 23HHZ, 23HGNZ, 23MXZ
37	Phenethyl alcohol	60-12-8	C_8_H_10_O	122.2	848	35.18	1894	1901	-	22HHZ, 22HGNZ, 22MXZ, 23HHZ, 23HGNZ, 23MXZ
38	2-Phenyl-2-propanol	617-94-7	C_9_H_12_O	136.2	917	31.03	1741	1759	-	22HGNZ, 22MXZ, 23HHZ, 23HGNZ, 23MXZ
39	2-Phenoxyethanol	122-99-6	C_8_H_10_O_2_	138.2	871	41.36	2140	2139	Pleasant	22HHZ, 22HGNZ, 22MXZ, 23HHZ, 23HGNZ, 23MXZ
40	1-Hexadecanol	36653-82-4	C_16_H_34_O	242.5	863	46.35	2386	2363	-	22HHZ, 22HGNZ, 22MXZ, 23HHZ, 23HGNZ, 23MXZ
	**Esters (2)**									
41	Pentyl hexanoate	540-07-8	C_11_H_22_O_2_	186.3	813	21.77	1496	1509	-	22HHZ, 22HGNZ, 22MXZ, 23HHZ, 23HGNZ, 23MXZ
42	gamma-Nonanolactone	104-61-0	C_9_H_16_O_2_	152.2	879	37.77	2006	2003	-	22HHZ, 22HGNZ, 22MXZ, 23HHZ, 23HGNZ, 23MXZ
	**Alkanes (3)**									
43	Dodecane	112-40-3	C_12_H_26_	170.3	859	9.42	1197	/	Gasoline like	22HHZ, 22HGNZ, 22MXZ, 23HHZ, 23HGNZ, 23MXZ
44	3-Methylundecane	1002-43-3	C_12_H_26_	170.3	811	13.19	1295	/	Floral, sweet, cooked vegetable	22HHZ, 22HGNZ, 22MXZ, 23HHZ, 23HGNZ, 23MXZ
45	Pentadecane	629-62-9	C_15_H_32_	212.4	859	21.29	1487	/	Mild odor	22HHZ, 22HGNZ, 22MXZ, 23HHZ, 23HGNZ, 23MXZ
	**Others (6)**									
46	2-Pentyl-Furan	3777-69-3	C_9_H_14_O	138.2	872	10.43	1226	1229	-	22HHZ, 22HGNZ, 22MXZ, 23HHZ, 23HGNZ, 23MXZ
47	2-Acetyl-1-pyrroline	85213-22-5	C_6_H_9_NO	111.1	810	14.29	1323	1323	Popcorn-like, sweet, pleasant, peanut, Cooked jasmine rice	22MXZ, 23MXZ
48	Naphthalene	91-20-3	C_1_0H_8_	128.2	890	29.61	1696	1707	Tar	22HHZ, 22HGNZ, 22MXZ, 23HHZ, 23HGNZ, 23MXZ
49	N,N-Dibutylformamide	761-65-9	C_9_H_19_NO	157.3	860	31.24	1750	1746	-	22HHZ, 22HGNZ, 22MXZ, 23HHZ, 23HGNZ, 23MXZ
50	Hexanoic acid	142-62-1	C_6_H_12_O_2_	116.2	877	33.84	1841	1854	Fatty, cheese, rancid	22MXZ, 23HGNZ, 23MXZ
51	Indole	120-72-9	C_8_H_7_N	117.2	876	47.26	2448	2448	Sour, fruit, floral, burnt	22HHZ, 22HGNZ, 22MXZ, 23HHZ, 23HGNZ, 23MXZ

“MW” represents molecular weight; “RT” refers to retention time; “RI” indicates the retention index calculated by the retention times of C9–C26 n-alkanes under the same condition; “RI (literature)” stands for the retention index from the NIST standard reference database (https://webbook.nist.gov/chemistry/, accessed on 22 May 2025); odor descriptions were found on the flavornet database (https://www.flavornet.org/flavornet.html, accessed on 22 May 2025) [2,25].

**Table 2:** Effects of treatments with different Se species on various VOCs detected in brown rice.

	Compounds	Variety (V)	Se Species (Se)	YEAR(Y)	V × Se	V × Y	Se × Y	V × Se × Y
66-25-1	Hexanal	170.44 ***	55.58 ***	2.1	80.09 ***	4.82 *	3.26 *	1.94
141-79-7	3-Penten-2-one, 4-methyl-	330.2 ***	187.19 ***	2.26	105.74 ***	14.9 ***	13.76 ***	1.67
111-71-7	Heptanal	545.17 ***	9.98 ***	2.84	11.92 ***	11.75 ***	2.68	6.86 ***
112-40-3	Dodecane	299.87 ***	37.94 ***	62.26 ***	94.68 ***	27.74	2.85	0.99
3777-69-3	2-Pentyl-Furan	24.13 ***	43.33 ***	1.16	8.8 ***	3.05	1.98	1.05
71-41-0	1-Pentanol	160.77 ***	17.33 ***	10.64 **	11.51 ***	0.21	2.33	3.01 *
124-13-0	Octanal	638.31 ***	19.42 ***	0.03	15.96 ***	1.39	0.13	1.88
1002-43-3	3-Methylundecane	180.87 ***	116.73 ***	1.69	38.34 ***	3.11	0.58	1.44
18829-55-5	(E)-2-Heptenal	178.28 ***	112.83 ***	1.37	20.24 ***	5.52**	0.57	2.92 *
85213-22-5	2-Acetyl-1-pyrroline	3030.46 ***	17.71 ***	188.84 ***	17.71 ***	188.84 ***	0.12	0.12
110-93-0	6-Methyl-5-hepten-2-one	141.7 ***	62.04 ***	29.08 ***	34.39 ***	10.21 ***	1.63	7.3 ***
123-42-2	4-Hydroxy-4-methyl-2-pentanone	553.85 ***	231.26 ***	0.92	130.63 ***	1.82	6.33 **	3.85 *
111-27-3	1-Hexanol	3415.48 ***	190.07 ***	254.04 ***	32.49 ***	317 ***	4.93 *	11.59 ***
124-19-6	1-Nonanal	2131.58 ***	116.73 ***	318.84 ***	329.13 ***	422.38 ***	44.13 ***	41.43 ***
1669-44-9	3-Octen-2-one	23.93 ***	49.96 ***	47.46 ***	10.43 ***	42.99 ***	3.09	2.5
2548-87-0	(E)-2-Octenal	68.56 ***	25.13 ***	100.67 ***	10.64 ***	50.41 ***	1.21	4.92 **
5989-33-3	(Z)-2-[(2R,5S)-5-Methyl-5-vinyltetrahydro-2-furanyl]-2-propanol	1231.56 ***	4.15 *	161.61 ***	4.43 **	450.13 ***	1.43	1.39
3391-86-4	1-Octen-3-ol	900.13 ***	24.94 ***	103.17 ***	20.71 ***	105.9 ***	16.86 ***	13.89 ***
111-70-6	1-Heptanol	521.89 ***	9.18 ***	81.62 ***	8.19 ***	70.13 ***	8.74 ***	2.18
34995-77-2	2-Furanmethanol,5-ethenyltetra	492.69 ***	6.12**	677.51 ***	6.74 ***	313.8 ***	0.9	2.01
693-54-9	2-Decanone	141.3 ***	1.32	162.68 ***	5.56**	121.88 ***	7.54 **	2.89 *
112-31-2	Decanal	317.56 ***	17.79 ***	205.28 ***	6.63 ***	133.71 ***	0.74	3.34 *
629-62-9	Pentadecane	188.71 ***	115.93 ***	41.44 ***	23.61 ***	27.23 ***	12.59 ***	7.97 ***
14309-57-0	3-Nonen-2-one	64.64 ***	68.34 ***	285.37 ***	172.84 ***	247.68 ***	20.81 ***	9.84 ***
100-52-7	Benzaldehyde	767.66 ***	149.17 ***	7461.2 ***	43.18 ***	442.5 ***	43.69 ***	2.69 *
540-07-8	Pentyl hexanoate	75.44 ***	9.58 ***	4.17 *	25.94 ***	4.24 *	0.61	1.14
111-87-5	1-Octanol	1137.11 ***	34.99 ***	342.46 ***	16.84 ***	392.76 ***	0.31	0.79
1604-28-0	3,5-Heptadien-2-one,6-methyl-	213.57 ***	2.75	22.64 ***	16.04 ***	8.31 **	0.11	8.04 ***
20019-64-1	2(5H)-Furanone, 5,5-dimethyl-	64.89 ***	11.81 ***	2.4	21.59 ***	3.26 *	5.79 ***	4.09 ***
529-20-4	2-Methylbenzaldehyde	5.69 **	7 **	111.91 ***	1.62	21.71 ***	0.49	1.01
22104-78-5	2-Octen-1-ol	11.58 ***	9.03 ***	244.33 ***	10.83 ***	73.71 ***	1.07	10.75 ***
3913-81-3	(E)-2-Decenal	551.07 ***	65.43 ***	333.94 ***	9.58 ***	226.7*	4.82	2.28
13019-16-4	2-Octenal, 2-butyl-	632.88 ***	305.96 ***	5.63 *	197.25 ***	2.75	0.34	1.15
98-55-5	alpha-Terpineol	470.2 ***	6.91**	470.2 ***	6.91 ***	470.2 ***	6.91 **	6.91 ***
91-20-3	Naphthalene	1.2	58.94 ***	65.06 ***	11.06 ***	2.33	4.31 *	2.64 *
617-94-7	2-Phenyl-2-propanol	264.12 ***	339.69 ***	161.12 ***	127.78 ***	106.32 ***	6.54 **	1.65
761-65-9	N,N-Dibutylformamide	240.62 ***	5.08 *	19.93 ***	15.65 ***	19.33 ***	2.77	4.36 **
112-30-1	1-Decanol	1.89	44.6 ***	914.43 ***	52.07 ***	20.73 ***	32.45 ***	44.88 ***
10486-19-8	Tridecanal	15.05 ***	0.19	11.3 **	3.42 *	1.86	1.44	0.11
3796-70-1	(E)-6,10-Dimethylundeca-5,9-dien-2-one	120.91 ***	34.22 ***	35.39 ***	3.14 *	30.95 ***	0.06	1.86
142-62-1	Hexanoic acid	668.42 ***	25.4 ***	207.55 ***	15.59 ***	89.42 ***	10.21 ***	7.93 ***
100-51-6	Benzyl alcohol	666.51 ***	50.81 ***	1078.45 ***	5.34 **	252.15 ***	2.12	17.27 ***
60-12-8	Phenethyl alcohol	290.24 ***	146.69 ***	85.23 ***	118.58 ***	9.26 ***	7.92 **	13.44 ***
104-61-0	gamma-Nonanolactone	47.55 ***	12.22 ***	6.67 *	3.8 *	3.52 *	1.72	0.2
616-45-5	2-Pyrrolidinone	221 ***	25.21 ***	39 ***	25.21 ***	39 ***	21.99 ***	21.99 ***
502-69-2	2-Pentadecanone,6,10,14-trimethyl-	1040.33 ***	101.47 ***	23.33 ***	9.51 ***	26.16 ***	4.71 *	2.02
122-99-6	2-Phenoxyethanol	16.85 ***	40.55 ***	2.29	22.41 ***	0.66	0.41	0.54
20189-42-8	3-Ethyl-4-methyl-1H-pyrrole-2,5-dione	544.69 ***	19.57 ***	143.54 ***	12.57 ***	117.01 ***	0	0.46
36653-82-4	1-Hexadecanol	166.69 ***	93.65 ***	148.26 ***	15.43 ***	113.71 ***	0.03	1.23
120-72-9	Indole	2532.55 ***	1475.51 ***	0.65	936.3 ***	73.43 ***	7.16 **	5.51 **
121-33-5	Vanillin	1198.31 ***	918.66 ***	1.24	116.51 ***	1.71	3.95 *	2.53

Variety (V): the different rice varieties; Se species (Se): the different Se treatments applied; YEAR (Y): the different planting years; V × Se: the interaction effect between variety and Se application; V × Y: the interaction effect between variety and year; Se × Y: the interaction effect between Se application and year; V × Se × Y: the three-factor interaction effect among variety, Se application, and year. The values here are F-values for each factor (V, Se, and Y) and their interactions. * *p* < 0.05; ** *p* < 0.01; *** *p* < 0.001 (Three-factor ANOVA).

## Data Availability

The original contributions presented in this study are included in the article/Supplementary Materials. Further inquiries can be directed to the corresponding author.

## Supplementary Materials

The following supporting information can be downloaded at: https://www.mdpi.com/article/10.3390/foods14193399/s1, Figure S1. Model validation metrics for OPLS-DA classification. Permutation tests (1000 permutations) showing R^2^Y and Q^2^ values for the Na2SeO3 treatment (A) and SeNPs treatment (B) respectively. Summary of R^2^X, R^2^Y, and Q^2^ values across different model components (p1, o1, o2, o3) for Na2SeO3 treatments (C) and SeNPs treatments (D). All models exhibited Q^2^ > 0.9 and *p* < 0.001, indicating robust predictive performance and no overfitting. Figure S2. The schematic diagram of field block structure. Table S1. The composition and concentration of volatile organic compounds detected in brown rice generated by different Se treatments of HHZ, MXZ and HGNZ in 2022. Table S2. The composition and concentration of volatile organic compounds detected in brown rice generated by different Se treatments of HHZ, MXZ and HGNZ in 2023. Table S3. The effect size of the bio-markers detected in Na_2_SeO_3_ and SeNPs treatments.
