# Supplementary figures and images for: Foliar-Selenium-Induced Modulation of Volatile Organic Compounds in Rice Grains: A Comparative Study of Sodium Selenite and Nano-Selenium
Source: Foods. 2025 Sep 30;14(19):3399. doi: 10.3390/foods14193399 (PMC12524218; doi:10.3390/foods14193399)

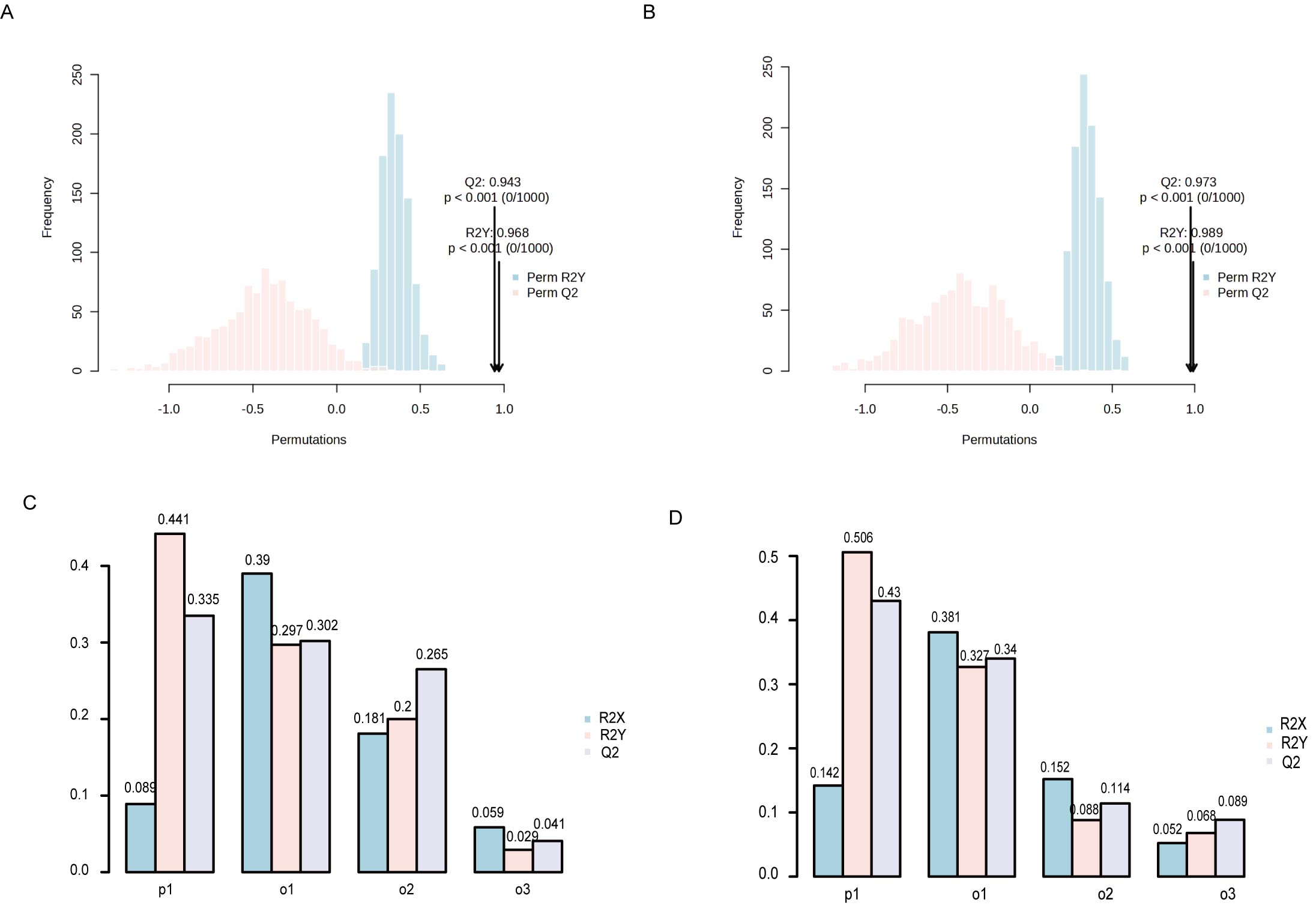

Supplement: Supplementary file 1 [file foods-14-03399-s001.zip › Supplementary Figure S1.jpg]

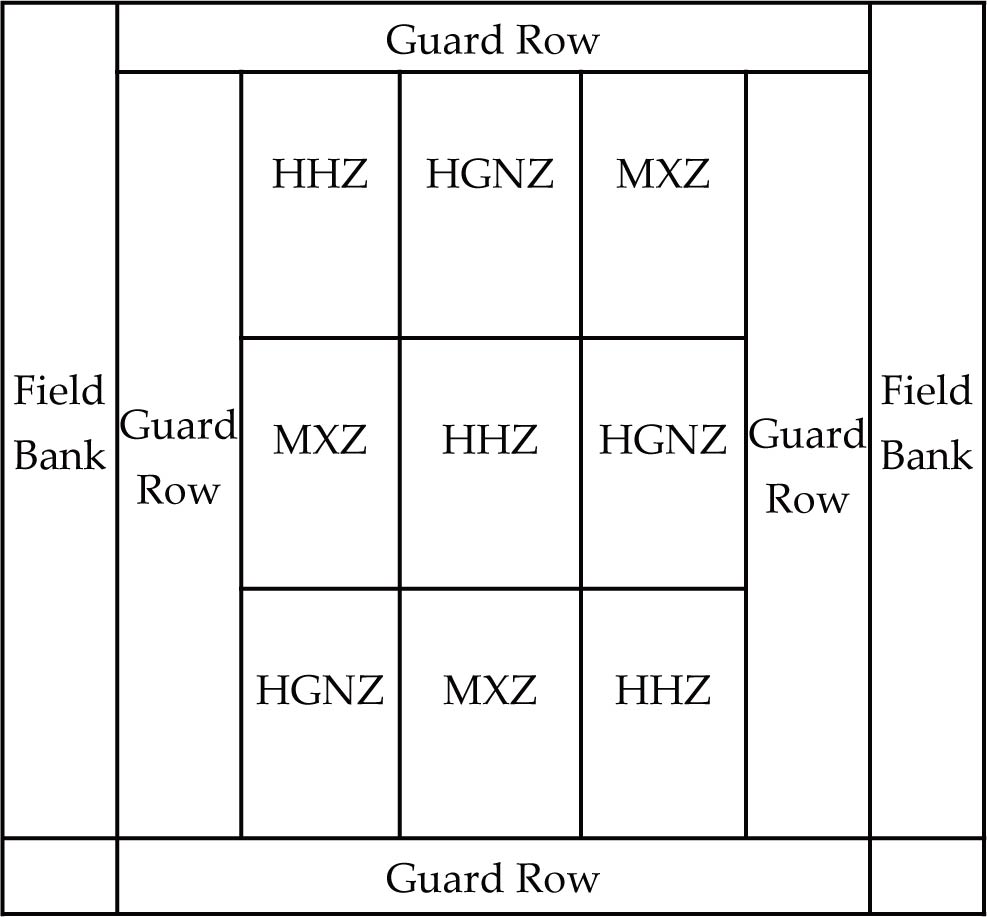

Supplement: Supplementary file 1 [file foods-14-03399-s001.zip › Supplementary Figure S2.jpg]
